# Cross-Cultural Adaptation, Reliability, and Validity of the Greek Version of the Fremantle Shoulder Awareness Questionnaire (FreSHAQ-GR) in Patients with Chronic Shoulder Pain

**DOI:** 10.3390/healthcare11182512

**Published:** 2023-09-11

**Authors:** George A. Koumantakis, Emmanouil Sifakis, Panagiotis Stathis, Spyridon Gigourtakis, Petros I. Tatsios, Eleftherios Paraskevopoulos, Maria Moutzouri, Sotiria Vrouva

**Affiliations:** 1 Laboratory of Advanced Physiotherapy, Physiotherapy Department, School of Health & Care Sciences, University of West Attica (UNIWA), 12243 Athens, Greece; phys18683136@uniwa.gr (E.S.); phys18683104@uniwa.gr (P.S.); ptatsios@uniwa.gr (P.I.T.); elparaskevop@uniwa.gr (E.P.); moutzouri@uniwa.gr (M.M.); 2 S. Gigourtakis Physiotherapy Practice, 71306 Heraklion, Greece; spgigourtakis@uth.gr; 3 Department of Physical Therapy, 401 Army General Hospital of Athens, 11525 Athens, Greece; svrouva@uniwa.gr

**Keywords:** shoulder pain, questionnaire, cross-cultural adaptation, validation, reproducibility, psychometric properties, body awareness

## Abstract

Shoulder pain is one of the most common musculoskeletal pathologies. The association of shoulder pain with impaired proprioception and bodily self-awareness called for the cross-cultural adaptation of the Fremantle Shoulder Awareness Questionnaire (FreSHAQ) in Greek. The FreSHAQ is a relatively new self-assessment 9-item scale for impaired somatic awareness in patients with shoulder pain. The study included 100 participants (54 women) between 20 and 80 years old, with chronic shoulder pain (>3 months duration), able to comprehend and speak Greek, and recruited from three private physical therapy practices. The cross-cultural adaptation process was followed to develop the Greek version of the questionnaire (FreSHAQ-GR). Further testing for construct validity via exploratory factor analysis and correlational analysis with the Shoulder Pain and Disability Index (SPADI), the Quick Disabilities of Arm, Shoulder, and Hand (QuickDASH), the pain catastrophizing scale (PCS), a pain intensity visual analog scale (PI-VAS) in the last week, and the demographic characteristics followed. Test–retest reliability of the FreSHAQ-GR in a sub-sample of patients (n = 25) was tested upon repeated completion of the scale after a week and examined with the intraclass correlation coefficient (ICC_2,1_), the standard error of the measurement (SEM), and the minimum detectable change (MDC) indices. The internal consistency of the scale was also assessed. The factor analysis revealed that the scale comprised one factor relevant to shoulder awareness but with fewer items (first 6 items) than the original FreSHAQ. Additionally, the FreSHAQ-GR (both versions) demonstrated statistically significant correlations (Spearman’s r) with participants’ age (r = −0.31/−0.29, *p* < 0.002), the PI-VAS (r = 0.54/0.52, *p* < 0.001), the SPADI total score and both of its subscales (r = 0.39–0.45/0.34–0.39, *p* < 0.001), the QuickDASH (r = 0.37/0.34, *p* < 0.001), and the PCS (r = 0.50/0.40, *p* < 0.001). The questionnaire’s internal consistency (Cronbach’s α/McDonald’s ω) was 0.833/0.827 for the 9-item and 0.871/0.867 for the 6-item FreSHAQ-GR. Test–retest reliability was excellent for both versions of the FreSHAQ-GR (ICC_2,1_(95% CI) = 0.97/0.98 (0.91–0.99/0.94–0.99), and with a low error margin (SEM = 1.31/0.94 and MDC_95%_ = 3.63/2.61). Therefore, the FreSHAQ-GR (6-item version) possesses very good measurement properties and can be administered to Greek-speaking patients with chronic shoulder pain.

## 1. Introduction

Shoulder pain incidence is ranked high among musculoskeletal-related disorders, with its incidence ranging from 7.7 to 62 per 1000 persons per year (median 37.8 per 1000 persons per year) [1]. Various factors such as personal (age, gender), work-related, environmental, economic, pain chronicity, central sensitization, and psychosocial overlay significantly affect the course and prognosis of the painful shoulder pathology [2,3,4,5]. Shoulder pain appears to increase after the age of 50 in the general population while also being prevalent in younger age groups, with 16 of 21 included studies in a recent systematic review reporting higher rates of pain in older participants; additionally, in the most physically active occupations over 50 years of age, incidence also increased [6]. The impact of physical limitations on both personal and professional aspects of daily life is significant for about 40% of individuals with chronic shoulder pain [7].

Most shoulder pathologies can be diagnosed at the first visit to primary care when a careful medical history is taken, combined with a proper physical examination [8,9,10]. The examination includes observation, palpation, and assessment of both passive and active range of motion (ROM), as well as resisted movements and specialized clinical examination tests [8,10]. The presence or absence of “red flags” in the medical history, denoting serious pathology, must also be assessed [9]. 

However, chronic shoulder pain develops in a large proportion of patients with acute shoulder pain complaints, irrespective of the cause of initial onset [1]. In chronic shoulder pain conditions, sensorimotor dysfunctions are present due to pathophysiological processing changes established along the peripheral pathways and the central nervous system processing centers [11,12]. The processes collectively contributing to sensorimotor dysfunction include motor deficits, altered sensory feedback, body representation cognitive distortions, altered multisensory cortical processing, and aberrant sensorimotor integration [12,13]. Additionally, several psychosocial factors have been linked to sensorimotor dysfunction, specifically the presence of central sensitization, in patients with chronic shoulder pain [14,15]. Current research studies surmise that the above-stated sensorimotor dysfunctions maintain pain chronicity [12], but various body-awareness re-training treatment strategies seem to reduce pain and disability symptomatology in patients with chronic shoulder pain [16,17,18]. Thus, detailed recording and understanding of sensorimotor adaptations in chronic pain is necessary to improve current models of care for chronic shoulder pain. 

The “body self-awareness” construct has been described as complex and multidimensional, and when considered maladaptive in certain states such as chronic pain, it manifests with cognitive distortions of painful body parts and is strongly influenced by mental and emotional processes [19]. Bodily self-awareness is not routinely included in the assessment of patients with chronic shoulder pain, and because of its importance in pain maintenance and resolution, it should be evaluated by relevant scales [20]. The Fremantle Back Awareness Questionnaire (FreBAQ) has been developed to specifically assess disturbed body self-awareness in relation to chronic back pain [21]. Further to this work, a shoulder-specific body self-awareness questionnaire was constructed and tested in a Japanese population with persistent shoulder pain, namely the Fremantle Shoulder Awareness Questionnaire (FreSHAQ) [20]. 

Therefore, the aim of this study was to conduct a cross-cultural adaptation of the FreSHAQ questionnaire in Greek, to determine its degree of reliability upon repeated administration and its structural validity by conducting exploratory factor analysis, and to determine its association with other shoulder pathology-related questionnaires.

## 2. Materials and Methods

### 2.1. Sample

The study was conducted on 100 adults aged between 20 and 80 years with a diagnosis of painful shoulder pathology lasting for more than three months who, after clinical evaluation, were positive in at least three of the following six clinical tests: (1) Hawkins impingement sign; (2) Neer’s impingement sign; (3) painful arc sign; (4) Jobe’s test; (5) Whipple’s test; (6) shoulder pain elicited by resistance testing during shoulder abduction or external rotation. In addition, participants were excluded based on the following criteria: (1) history of shoulder surgery or planned shoulder surgery; (2) history of fracture, dislocation, or degenerative disease of the shoulder joint; (3) active shoulder range of motion <90 degrees of flexion or abduction or <0 degrees of external rotation; (4) cervical radiculopathy or examination that caused shoulder pain/reduced range of motion after repetitive neck movements; and (5) other serious orthopedic problems [20].

All participants spoke and understood the Greek language and were referred by the treating physician to either of three private physiotherapists (two in Athens and one in Crete). All data obtained for this study were collected before the start of their treatment.

### 2.2. Ethical Considerations

The protocol of this study was approved by the Ethics Committee of the University of West Attica, Athens, Greece (approval no: 53346/07.06.2022), according to the Declaration of Helsinki. All participants were administered a detailed information sheet describing the aims and purposes of this study, and those who agreed to participate filled out and signed a relevant consent form prior to their inclusion in the study.

### 2.3. Study Design

This study comprised two parts: (a) the cross-cultural adaptation process of the FreSHAQ scale and (b) the validation of this scale in the Greek (target) language.

### 2.4. Procedures

#### 2.4.1. Cross-Cultural Adaptation Process

Permission was obtained from the developers of the FreSHAQ scale (Prof. Benedict Martin Wand and Dr. Tomohiko Nishigami) for the cross-cultural adaptation of the scale in Greek. The cross-cultural adaptation was performed based on the methodology previously described [22,23]. Specifically, two “forward” translations from English to Greek were independently completed by two bilingual translators (a healthcare professional and one without clinical experience in the health field). The two translations were then synthesized into one, with any minor discrepancies discussed and resolved, resulting in a preliminary version of the scale in Greek. Then, the “back translation” process of the preliminary Greek version into English was performed by two Greek-speaking language experts whose mother tongue was English (a healthcare professional and a non-health professional) without having access to the original version of the scale. After the back-translation process was completed, the review committee, consisting of a methodologist (the main researcher), a healthcare professional, and all the translators, evaluated all versions of the FreSHAQ in English and Greek to ensure proper format, wording, grammar, and meaning. Any inconsistencies were discussed and resolved by consensus, leading to the pre-final Greek version of the scale, the FreSHAQ-GR. To ensure the conceptual and semantic equivalence of the pre-final FreSHAQ-GR, it was initially pilot tested in 10 patients with a painful shoulder pathology. Those patients and the committee members were asked to rate the questionnaire instructions and items using a dichotomous scale (clear/unclear), and if any were unclear, participants were asked how these could be rephrased to become clearer. If any part of the questionnaire was deemed unclear by at least 20% of the pilot-testing participants, it had to be re-evaluated [23]. Finally, the questionnaire was submitted for content equivalence evaluation (content-related validity) to the committee members using a 4-level Likert scale (1 = not relevant; 2 = unable to assess relevance; 3 = relevant but minor alteration required; and 4 = very relevant and concise). Items classified as 1 or 2 had to be revised [23]. The content validity index at the item level (I-CVI) and at the scale level (S-CVI) was calculated, the latter using the averaging calculation method [24].

#### 2.4.2. FreSHAQ-GR Validation Process

The next part of the study was the full application of the final version of the translated questionnaire to patients. The questionnaires were given to patients with painful shoulders in two physical therapy practices in Athens and one in Crete after the consent of their respective scientific directors had first been secured. 

Data collection included a questionnaire of patient demographic details (age, sex, height, weight, BMI, marital and work status, educational level), the cross-culturally adapted Greek version of the FreSHAQ (FreSHAQ-GR), a Pain Visual Analogue Scale over the past week on average (P-VAS), the Quick Disabilities of the Arm, Shoulder and Hand questionnaire in Greek (QuickDASH-GR), the Shoulder Pain and Disability Index in Greek (SPADI-GR), and the Pain Catastrophizing Scale in Greek (PCS-GR). A quarter of the participants (n = 25) filled in the FreSHAQ-GR again after 5–7 days without any intervening physical therapy session between those two occasions and at the same time of day to assess the test–retest reliability of the scale. All scales were administered to patients in paper format by either of the two student physical therapists at the time (ES and PS). 

#### 2.4.3. The Fremantle Shoulder Awareness Questionnaire (FreSHAQ)

The FreSHAQ questionnaire is a relatively new assessment tool [20] that resulted from modifying the Fremantle Back Awareness Questionnaire (FreBAQ) [21]. The FreSHAQ aims to assess impairments in proprioception, altered sensations regarding the size and shape of the body part in pain, and motor control discrepancies as patients are asked to answer questions targeting those domains. Items 1–3 of the FreBAQ (and the FreSHAQ also) were adopted from the Galer and Jensen questionnaire [21,25], denoting cognitive and motor neglect of the painful part. Proprioception impairment is assessed by items 4 (motion perception impairment) and 5 (repositioning impairment), whereas items 6–9 relate to impaired body perception (size and shape) of the painful body part [21]. Therefore, the FreSHAQ consists of 9 questions, which refer to the person’s perception of their shoulder, with each question scored on a 5-point Likert scale giving 5 possible answers, from 0 (never) to 4 (always). Its overall score ranges from 0–36. Higher scores are associated with greater levels of disturbed bodily self-awareness.

The FreSHAQ was studied for the first time in a Japanese population of patients with persistent shoulder pain and was found to be unidimensional (fit statistics from Rasch analysis), not include inconsistent items (person fit analysis), and have excellent reliability upon repeated administration [20]. Additionally, it is a quick, simple, and easy-to-comprehend assessment tool for patients with painful shoulder pathology.

#### 2.4.4. Pain Intensity Visual Analog Scale (PI-VAS)

The visual analog scale (VAS) is widely used in adults with different pathologies as a measure of their pain intensity [26]. The pain intensity VAS (PI-VAS) scale usually consists of a 100 mm-long horizontal line with word descriptions at each end, i.e., no pain at all (left) and very severe pain (right). The patient marks the line at the level where, in their opinion, their pain intensity is best described. The VAS scale score is calculated by measuring in millimeters from the left end of the line to the point marked by the patient [26]. Moderate to good levels of reliability of the PI-VAS scale in chronic musculoskeletal pain [27]. As different recall periods of the scale have been used [26,27], we chose to use the “average pain intensity in the last week” recall period.

#### 2.4.5. Quick Disabilities of the Arm, Shoulder, and Hand (QuickDASH)

A shortened version of the Disabilities of the Arm, Shoulder, and Hand (DASH) is the QuickDASH, where instead of 30 items related to physical function, symptoms, and social functioning, only 11 items are used to measure physical function and symptoms in people with any musculoskeletal upper extremity disorder [28]. At least 10 of the 11 items must be completed to calculate a QuickDASH score. Each item has 5 response options (1—no difficulty, 2—mild difficulty, 3—moderate difficulty, 4—severe difficulty, 5—no ability) from which the scale scores are calculated, ranging from 0 (no dysfunction) to 100 (most severe dysfunction) [28]. It is a valid and reliable measure of shoulder pain conditions and is the most widely used assessment tool for shoulder pathologies [29]. The QuickDASH questionnaire has been cross-culturally adapted and validated in Greek [30].

#### 2.4.6. Shoulder Pain and Disability Index (SPADI)

The Shoulder Pain and Disability Index (SPADI) is a self-administered questionnaire designed to assess pain and disability under various activities with a recall period of one week in patients with shoulder dysfunction. It consists of 13 items, with the first 5 assessing pain and the remaining 8 assessing disability. Patients answer each item according to the degree of pain felt and difficulty in performing activities on a numerical rating scale ranging from 0 (for no pain and no difficulty) to 10 (for maximum pain and great difficulty). The final score is obtained by summing up the scores per item and calculating a percentage (%) for the total score and the two subscales. Average completion time 5–10 min. The SPADI was found to be particularly responsive to change and easy to complete, with very good validity [29]. It has also been cross-culturally adapted and validated in a Greek population [31].

#### 2.4.7. Pain Catastrophizing Scale (PCS)

The Pain Catastrophizing Scale (PCS) questionnaire measures the degree of negative and catastrophic thoughts in relation to actual, perceived, or anticipated pain experience [32]. It consists of 13 questions, each scored on a 5-point Likert scale, with answers ranging from 0 (never) to 4 (always). The total score ranges from 0 (no perception of pain catastrophizing) to 52 (high perception of pain catastrophizing). It demonstrates excellent psychometric properties, offering a multidimensional measurement of pain-related catastrophizing [33]. The PCS has been cross-culturally adapted and validated in Greek [34].

### 2.5. Statistical Analysis

Statistical analysis was performed with the IBM Statistical Package for the Social Sciences (IBM SPSS v.28). All continuous variables were analyzed for normality of distribution with the Kolmogorov–Smirnov test. The descriptive statistics of participants and their scores in the FreSHAQ-GR, PI-VAS, SPADI, QuickDASH, and PCS scales were presented, depending on the distribution of each variable. For variables that were normally distributed, the mean (SD), maximum, and minimum values were reported; for those that were not, the median and interquartile range (IQR) values were additionally reported.

Construct validity expresses the degree to which the questionnaire is consistent with the theoretically generated assumptions about the concepts being measured [35]. Two aspects of construct validity of the FreSHAQ-GR were studied: By examination of the factor structure of the questionnaire via exploratory factor analysis (EFA) [36] and by testing for associations of the FreSHAQ-GR with other relevant patient-reported outcomes (PROs) [23]. For EFA, the minimum acceptable required sample size usually requires 10 participants per questionnaire item [23,36]; however, some suggest that for a stable solution to be granted, the sample should include a minimum of 100 individuals and factors comprising 3–4 strong items (with loadings of 0.70 or greater) [37,38]. Therefore, both of these recommendations were considered regarding the minimum acceptable sample size. EFA was assessed by using the principal components analysis (PCA) method and additionally applying the Varimax rotation with the Kaiser normalization method [36]. The Bartlett test of sphericity and the Kaiser–Meyer–Olkin (KMO) measure of sampling adequacy were used to examine the sufficiency of the population used as a sample for this study. The number of factors to be extracted was determined using parallel analysis, by extracting eigenvalues from random data sets that parallel the actual data set relating to the number of cases and variables [39], as using only the Kaiser criterion (eigenvalues > 1) for this purpose has been criticized for factor over-extraction [38,39]. A sound model was considered one in which at least 50% of overall scale variance could be explained by the items, communalities no less than 0.40, no factor loading less than 0.32, no item loaded across more than one factor at a level more than 0.32, and factors consisting of at least 3–5 items with strong loadings (>0.70) on one factor only [38,40].

To additionally test the construct validity, correlations with relevant PROs to the patients’ condition (PI-VAS, SPADI, QuickDASH, and PCS) were assessed. Correlations were rated as very strong (0.90–1.00), strong (0.70–0.89), moderate (0.4–0.69), weak (0.1–0.39), or negligible (0.0–0.10) [41]. The significance level for all comparisons was initially set at 0.05. However, due to several correlation tests performed, the significance level was adjusted according to the Holm–Bonferroni method [42]. The adjusted a-level was calculated at a = 0.0083 based on the six correlations of interest between the FreSHAQ and the PI-VAS, SPADI total, and 2 subscales, QuickDASH, and PCS. Therefore, to achieve 80% power with a relatively weak correlation level of r = 0.35, the required sample size would be n = 94 participants. All relevant calculations were performed with an online program for computing power and sample size for correlational designs (https://sample-size.net/correlation-sample-size/ (accessed on 21 August 2023)) [43].

Internal consistency reliability is an indicator that reveals whether the different questionnaire items measure various facets of the same characteristic and was estimated with the Cronbach’s α coefficient [35] and additionally with the McDonald’s Omega (ω) coefficient, considered a better option for items that present with a skewed distribution [44], with values above 0.7 for both coefficients considered acceptable [45]. The between-day test–retest reliability was calculated using the two-way mixed effects absolute agreement single rater intraclass correlation coefficient (ICC2,1) [46], the standard error of the measurement (SEM), and the minimum detectable change (MDC95%) [47]. ICCs less than 0.5 were considered poor, 0.5–0.75 moderate, 0.75–0.90 good, and greater than 0.90 excellent [46]. The SEM and MDC95% are indicators of the error level inherent within the measurement, with the MDC representing the smallest amount of change that can be detected between two time periods that is not due to measurement error [35,47].

Floor/ceiling effects were considered present if the total score of the FreSHAQ-GR scale corresponded to either of the extreme scale values (0 or 36) for more than 15 participants (15% of the sample) [45].

## 3. Results

### 3.1. Cross-Cultural Adaptation of the FreSHAQ-GR Questionnaire

No particular problems were met during the cross-cultural adaptation process of the FreSHAQ, except for questions 7 and 8, in which there was difficulty in capturing the phrases “larger than it appears” and “smaller than it appears”, respectively, in the Greek language with clarity and precision, which was appropriately dealt with by consensus. During pilot testing of the pre-final version, the experts’ committee and the patients rated all items as clear. Furthermore, the committee found all questions very relevant to the concept of shoulder bodily self-awareness (I-CVI = 1 and S-CVI/Ave = 1). Therefore, the finalized version of the FreSHAQ-GR (Supplementary Materials) was administered to the patient group for the validation process.

### 3.2. Descriptive Statistics

The Kolmogorov–Smirnov test has revealed that height, pain duration, and most of the questionnaire scores (FreSHAQ-GR, PI-VAS, SPADI-Pain Subscale, QuickDASH, and PCS) did not follow a normal distribution. Only the SPADI-Disability Subscale and SPADI-Total Score data followed a normal distribution. Therefore, descriptive statistics were presented both as means (standard deviations, SD) and medians (interquartile ranges, IQR) for demographic characteristics (Table 1) and questionnaire scores (Table 2 and Table 3). All correlational analyses were performed with a non-parametric test (Spearman’s correlation coefficient).

A positively skewed distribution was noted for the Quick-FAAM-GR Total Score (0.584), and the response frequencies per item of the FreSHAQ-GR confirm this, indicating that, depending on the item, 44–84% of participants reported “never feeling like that”, apart from item 2 (Table 3). However, it was also evident that participants with persistent shoulder pain also reported body awareness impairments, in varying frequencies and degrees of severity, between 16 and 74% depending on the item of the FreSHAQ-GR. Out of the 100 participants with shoulder pain, 54 were women. Participants had suffered persistent shoulder pain either on their right (n = 67) or left shoulder (n = 33) for over 3 months. For the FreSHAQ-GR, no floor or ceiling effects were observed; only seven participants’ total score on the FreSHAQ-GR was 0, and for none was 36.

### 3.3. Construct Validity of the FreSHAQ-GR

The data collected were suitable for factor analysis, as attested by Bartlett’s test of sphericity, which was significant (chi-square = 380.62, df = 36, *p* < 0.001), and the value of the Kaiser–Meyer–Olkin measure of sampling adequacy, which was acceptable (0.795) [36]. The initial factor structure, including all 9 items using the PCA method and based on the Kaiser criterion (eigenvalue > 1), revealed a two-factor solution with an eigenvalue for factor 1 (consisting of 7 items) of 4.13 and an eigenvalue of 1.39 for factor 2 (consisting of 2 items), accounting for 61.30% of the variance, with the first factor accounting for 45.89% of the total variance. The communalities of the 9 items ranged from 0.41 to 0.84, with item 8 having the lowest communality. The factor loadings of the items from the component matrix ranged from 0.62 to 0.91 (Table 4). However, the parallel analysis indicated that a single factor should be extracted [39]. Additionally, a factor consisting of two items is considered unstable [38,40].

Therefore, we further evaluated the factor structure of the FreSHAQ-GR with the items of factor 2 of the initial solution removed, thereby bringing a 7-item version forward. For the single-factor 7-item solution, Bartlett’s test of sphericity was significant (chi-square = 323.54, df = 21, *p* < 0.001), and the KMO measure of sampling adequacy was high (0.842) [36]. This shortened version resulted in an improvement in the variance explained by a single-factor solution (56.37%). However, the communality for item 8 was 0.32 (Table 5), well below the 0.40 value suggested as the lower acceptable value [38,40]. Upon removal of this item, a 6-item single-factor solution was examined.

For the 6-item single-factor solution, Bartlett’s test of sphericity was significant (chi-square = 297.38, df = 15, *p* < 0.001), and the KMO measure of sampling adequacy was high (0.828) [36]. Communalities ranged between 0.49 and 74 and factor loadings between 0.70 and 0.84. This single factor seemed to provide an even better solution, as it accounted for 61.44% of the total variance (Table 6).

Construct validity was assessed via correlations of the FreSHAQ-GR with other patient demographic characteristics, and PROs demonstrated highly statistically significant Spearman’s correlations (*p* < 0.001) for both the 9 and 6-item versions of the FreSHAQ-GR with age, the PI-VAS, the SPADI disability rating scale and both its subscales, the QuickDASH Disability Scale, and the PCS (*p* < 0.001). Finally, the correlation between the 9- and 6-item versions of the FreSHAQ-GR was also high (Table 7).

### 3.4. Reliability

Internal consistency was measured by using Cronbach’s α coefficient. The overall (9-item) Cronbach’s α coefficient was 0.833 (9-item FreBAQ-GR) and for Factor 1 (6-item) was 0.871, both considered very good [35]. The respective McDonald’s ω coefficients were 0.827 and 0.867, almost identical to Cronbach’s α coefficients. However, Cronbach’s α for the initially identified Factor 2 (consisting of items 7 and 9) was 0.67 (considered fair), and the respective McDonald’s ω could not be calculated as this factor consisted of only two items.

The test–retest intra-rater relative reliability measured with the ICC_2,1_ (95% CI) values was excellent (>0.90), and the absolute reliability indices quantifying the amount of test–retest error (SEM and MDC_95%_) were sufficiently low, both for the 9- and the 6-item versions of the FreSHAQ-GR (Table 8) [35]. Specifically, in terms of absolute reliability, the SEM and MDC_95%_ values were at acceptable levels relative to the range of values of the 9-item questionnaire (0–36) and the 6-item (0–24), and if interpreted as percent error for the 9-item FreSHAQ-GR in relation to a grand mean of 11.44, these amounted, respectively, to (1.31/11.44) × 100 = 11.45% and (3.63/11.44) × 100 = 31.70%, while for the 6-item FreSHAQ-GR, respectively, to 10.17% and 28.25%. SEM and SDD indices were not measured in the FreSHAQ-J.

## 4. Discussion

Cross-cultural adaptation is an integral and important part of clinical research, as valid, reliable, and easy-to-use PROs are required for effective patient treatment and follow-up [23,45]. The cross-cultural adaptation of the FreSHAQ in the Greek language was necessary as it is the only tool that assesses bodily self-awareness in patients with chronic shoulder pain [20], resulting in an enhancement of the clinical practice monitoring of both Greek physiotherapists and medical doctors. Impaired body awareness forms part of the sensorimotor disturbance cascade present in persistent musculoskeletal pain conditions [13]. As sensorimotor disturbance associated with chronic pain may prolong chronicity [12], PROs with valid psychometric properties are required for effective monitoring of manifestations [19] and treatment approaches [48] related to body awareness.

The FreSHAQ was previously only available in its Japanese version (FreSHAQ-J) [20]. For comparability, the age range and inclusion–exclusion criteria of participants were identical to those in the Japanese version. Specifically, the population used in the current study consisted of patients with persistent chronic shoulder pain of more than 3 months duration, referred by orthopedic physicians for physical therapy in three different locations. After a standard clinical examination, participants had to be positive in three orthopedic shoulder tests to be included. Pain duration on average (±SD) was similar between participants of the current study (10.2 ± 19.7 months) and the Japanese population (12.8 ± 13.4 months) studied.

The cross-cultural adaptation process of the FreSHAQ-GR was seamlessly executed, with the scale being rated as clearly phrased and all its items rated as relevant to the patients’ condition and symptomatology. Moreover, in the present study, no “floor” or “ceiling effects” were present for the total scale score, similar to the study by Nishigami et al. [20], but for all individual items of the scale, floor effects were present. This suggests that the questions should not be evaluated separately, but the final score of the questionnaire should be considered. The general pattern of response frequency per item (Table 3) presented similarities between this and the Japanese validation study, with an increased percentage of participants rating “never” or “rarely” all of the scale items. In the current study, item 2 was rated as “never” the lowest (26%), whereas in the Japanese study, item 1 (23.2%) and item 9 (8.9%) were rated as “never”. However, all other ratings per item (“occasionally”, “often”, and “always”) were selected by a considerable number of participants, and the total scale score was similar between the two populations. Perhaps the similarities in the response frequency pattern reflect the between-population parity in the pathology characteristics, severity, and duration between the two sample populations. In addition, possibly the clearly phrased instructions and items of the scale in both instances resulted in a similar understanding of the concepts examined by participants in both studies.

Rasch analysis was previously conducted for the FreSHAQ-J, revealing the scale’s unidimensionality; however, EFA has not been conducted. In this study, we conducted an EFA of the FreSHAQ-GR with two factors initially extracted: Factor 1 comprising questions 1–6 and 8, and factor 2 containing questions 7 and 9. However, parallel analysis indicated that a single factor should be extracted. Moreover, the internal consistency reliability of McDonald’s ω coefficient of the second factor could not be calculated as it consisted of only two items. Then, item 8 was also removed as it presented low communality. The single factor finally extracted collectively explained 61.44% of the variability in shoulder self-awareness. The FreSHAQ-J was based on the FreBAQ scale, comprising nine items, the first three relating to cognitive and motor neglect, the following two relating to proprioception impairment, and the last four depicting impaired body perception (size and shape) of the painful body part [20,21]. However, in our study, items 7–9 could not be included together with the first six items in a single-factor solution. It seems that of the four items of the FreSHAQ-J representing impaired body perception, only item 6 was retained in the FreBAQ-GR, while items 7 and 8 (the painful shoulder feels “larger than it appears” and “smaller than it appears”) and item 9 (“my shoulders feel different between left and right—in terms of size and shape”) were scored differently in our sample, for reasons that could be related to the comprehensibility of those items or due to the fact that participants did not feel their shoulder the way these items described it. Especially for items 7 and 8, which state two opposing conditions, it can be more readily realized why there was no affinity between the two unless the same subjects felt their shoulder on occasion larger or smaller than it appears. As there is currently no similar data from other cultures and populations in chronic shoulder pain, and also because in the FreSHAQ-J no exploratory factor analysis was used, it is difficult to directly compare and interpret these findings, although a single factor solution was also reached. Our results are perhaps comparable to a generic version of the Fremantle Body Awareness Questionnaire recently proposed, where a 6- and 7-item solution was also found [49]; however, in that research, a population with mixed musculoskeletal chronic pain pathologies was used.

To further examine the construct validity of the FreSHAQ questionnaire, the degree of association with patient demographic characteristics but most importantly with other scales examining the clinical status of patients showed highly significant correlations (*p* < 0.001) for both the 9 and 6-item versions of the FreSHAQ-GR with age, the PI-VAS, the SPADI, the QuickDASH, and the PCS. These results confirm previous findings of significant associations between the FreSHAQ-J and pain VAS (r = 0.2), the QuickDASH (r = 0.49), and the PCS (r = 0.38) [20]. We chose to examine associations with the SPADI scale in addition to the QuickDASH, as the former separates into two subscales relating to pain and disability, but also as the two scales measure slightly different components of shoulder-specific disability and function [50]. Since all scales are highly associated with the FreSHAQ-GR, it can be surmised that there is a significant inter-relationship between patient clinical status and bodily self-awareness. However, there was no association present between the duration of symptoms and the FreSHAQ-GR.

The internal consistency of the FreSHAQ-GR measured with two relevant indices (Cronbach’s α and McDonald’s ω) was very good for both the 9- and 6-item versions, with comparable values between the two indices. For the 9-item FreSHAQ-GR, the internal consistency was higher compared to that of the FreSHAQ-J (α = 0.71), with the latter value also acceptable.

The test–retest reliability of the questionnaire with a time interval between 5 and 7 days in 25 participants was excellent for both the 9 and 6-item versions of the FreSHAQ, with an ICC2,1(95% CI) = 0.97/0.98 (0.91–0.99/0.94–0.99), respectively. In comparison, the test–retest reliability of FreSHAQ-J was also very good, demonstrating an ICC3,1(95% CI) = 0.84 (0.70–0.92) [20]. Furthermore, the absolute indices of reliability, indicating the inherent error in measurement (SEM and MDC_95%_), were at acceptable levels relative to the range of values of the questionnaire. The MDC_95%_ = 3.63 for the 9-item and MDC_95%_ = 2.61 for the 6-item. FreSHAQ-GR in particular represents the smallest amount of true change in the clinical condition of a patient, meaning that scores ±4 for the 9-item and ±3 for the 6-item FreSHAQ-GR of a patient’s initial scores are probably due to a genuine improvement (or deterioration) in their clinical condition.

Some limitations present in this study were the fact that there were no other cross-cultural adaptations of the FreSHAQ-J, so it was not possible to collect and compare data from other similar studies with the results from the present study. The number of participants utilized satisfied the minimum criteria for EFA analysis. Moreover, the number of participants included in the test–retest reliability study was a quarter of the total sample, which is adequate for this type of investigation [35]. Lastly, the responsiveness of the FreSHAQ-GR scale was not tested in this study.

As far as future investigations are concerned, there is a need for further research in somatic self-awareness [12,20]. The responsiveness of the FreSHAQ-GR can be tested in populations with persistent shoulder pain that are treated with either newly emerging movement-based rehabilitation approaches [51] or more traditional ones [52], all of which target body awareness from a different perspective. These treatment approaches may require an assessment of their effectiveness not only on pain control and functional improvement but also on body awareness improvement, as this latter component may prove to have an important mediation effect in the rehabilitation process [13]. Furthermore, a potential avenue for future research is validating the FreSHAQ using methods grounded in item response theory, where response patterns are analyzed instead of item summation [53]. Additionally, confirmatory factor analysis [49] should be an area of future investigation for the FreSHAQ-GR. Finally, we would highlight the significance of validating the FreSHAQ across diverse age groups, such as young and elderly individuals; thus, conducting multigroup analyses in future studies could also be explored.

## 5. Conclusions

The cross-cultural adaptation of the FreSHAQ scale in Greek for patients with chronic shoulder pain was completed, presenting sufficient face and content validity. This was the first cross-cultural adaptation of this scale in another language and culture in relation to the original validation study. The results of the current study confirm that the scale possesses very good psychometric properties, comparable to the original scale, with very good internal consistency and excellent test–retest reliability. Regarding its construct validity, it correlates significantly with various indices of patient clinical status, and factor analysis revealed a one-factor solution for the FreSHAQ-GR, however, comprising the first six items of the original FreSHAQ-J scale. It is therefore recommended for further use in the clinical and research environment involving Greek-speaking patients with pain and functional limitations related to chronic shoulder pain conditions.

## Figures and Tables

**Table 1:** Descriptive statistics of the demographic characteristics (n = 100).

	Mean (SD)	Min–Max	Median (IQR)
Age (y)	54.37 (15.80)	20.00–80.00	52.00 (24.00)
Height (cm)	169.08 (9.79)	150.00–191.00	170.00 (15.00)
Body mass (kg)	76.17 (14.18)	43.00–106.00	78.00 (22.00)
Body mass index (kg/m^2^)	26.55 (4.03)	17.90–37.17	26.35 (6.24)
Pain duration (months)	10.17 (19.72)	3.00–132.00	4.00 (5.75)

**Table 2:** Descriptive statistics of the questionnaires (n = 100).

	Mean (SD)	Min–Max	Median (IQR)
FreSHAQ-GR	9.79 (7.50)	0.00–32.00	9.00 (13.00)
PI-VAS	5.31 (2.17)	0.00–10.00	5.00 (3.00)
SPADI-GR-Pain Subscale	59.76 (24.73)	0.00–98.00	60.00 (43.00)
SPADI-GR-Functional Subscale	47.38 (25.67)	0.00–96.30	47.50 (38.80)
SPADI-GR-Total	52.13 (24.23)	0.0–96.90	53.85 (39.60)
QuickDASH-GR	44.64 (20.19)	0.0–97.70	43.19 (26.73)
PCS-GR	19.34 (11.20)	0.00–44.00	17.00 (16.00)

FreSHAQ: Fremantle Shoulder Awareness Questionnaire, SPADI: Shoulder Pain and Disability Index, QuickDASH: Quick Disabilities of the Arm, Shoulder, and Hand, PCS-GR: Pain Catastrophizing Scale.

**Table 3:** Response frequencies per item and mean (SD) scores per item and total of the FreSHAQ-GR.

Item	Never N (%)	Rarely N (%)	Occasionally N (%)	Often N (%)	Always N (%)	Mean (SD)	Median (IQR)
1. My sore shoulder feels as though it is not part of the rest of my body	46	22	15	10	7	1.10 (1.28)	1.00 (2.00)
2. I need to focus all my attention on my sore shoulder to make it move the way I want it to	26	11	28	20	15	1.87 (1.40)	2.00 (3.00)
3. I feel as if my sore shoulder sometimes moves involuntarily, without my control	64	14	9	8	5	0.76 (1.21)	0.00 (1.00)
4. When performing everyday tasks I don’t know how much my sore shoulder is moving	44	14	22	16	4	1.22 (1.28)	1.00 (2.00)
5. When performing everyday tasks I am not sure exactly what position my sore shoulder is in	50	13	16	17	4	1.12 (1.30)	0.50 (2.00)
6. I can’t perceive the exact outline of my sore shoulder	50	15	12	14	9	1.17 (1.41)	0.50 (2.00)
7. My sore shoulder feels larger than it appears	54	13	11	14	8	1.09 (1.39)	0.00 (2.00)
8. My sore shoulder feels smaller than it appears	84	10	1	2	3	0.30 (0.85)	0.00 (0.00)
9. My shoulders feel different between left and right (in terms of size and shape)	49	9	16	13	13	1.32 (1.50)	1.00 (3.00)
Total Score						9.79 (7.50)	9.00 (13.00)

**Table 4:** Eigenvalues, communalities, and component matrix (rotated factor loadings) of exploratory factor analysis including all 9 items of the FreSHAQ-GR.

Factor	Initial Eigenvalues			Communalities	Component Matrix	
	Total	% of Variance	Cumulative %		Factor 1	Factor 2
1	4.13	45.89	45.89	0.65	0.77	
2	1.39	15.42	61.30	0.48	0.64	
3	0.86	9.56	70.86	0.60	0.77	
4	0.78	8.69	79.56	0.71	0.84	
5	0.64	7.14	86.70	0.71	0.84	
6	0.39	4.30	91.01	0.49	0.67	
7	0.31	3.46	94.47	0.84		0.91
8	0.28	3.07	97.54	0.41	0.62	
9	0.22	2.46	100.00	0.63		0.77

**Table 5:** Eigenvalues, communalities, and component matrix of exploratory factor analysis including 7 items of the FreSHAQ-GR.

Factor	Initial Eigenvalues			Communalities	Component
	Total	% of Variance	Cumulative %		Matrix
1	3.95	56.37	56.37	0.64	0.80
2	0.80	11.50	67.87	0.48	0.69
3	0.75	10.69	78.56	0.60	0.78
4	0.64	9.18	87.74	0.71	0.84
5	0.34	4.83	92.57	0.70	0.84
6	0.30	4.25	96.82	0.49	0.70
7	0.22	3.18	100.00	0.32	0.56

**Table 6:** Eigenvalues, communalities, and component matrix of exploratory factor analysis including the first 6 items of the FreSHAQ-GR.

Factor	Initial Eigenvalues			Communalities	Component
	Total	% of Variance	Cumulative %		Matrix
1	3.69	61.44	61.44	0.63	0.80
2	0.78	13.04	74.47	0.49	0.70
3	0.64	10.72	85.19	0.62	0.79
4	0.37	6.10	91.29	0.74	0.86
5	0.30	4.96	96.26	0.71	0.84
6	0.22	3.74	100.00	0.49	0.70

**Table 7:** Spearman’s correlation coefficients between the 9-item and 6-item FreSHAQ-GR and age, PI-VAS, SPADI-GR (subscales and total score), QuickDASH, and PCS-GR (n = 100).

	FreSHAQ-GR (9-Item)	FreSHAQ-GR (6-Item)
Age	−0.31 **	−0.29 **
PI-VAS	0.54 **	0.52 **
SPADI-GR-Pain Subscale	0.39 **	0.34 **
SPADI-GR-Functional Subscale	0.45 **	0.39 **
SPADI-GR-Total	0.45 **	0.39 **
QuickDASH-GR	0.37 **	0.34 **
PCS-GR	0.50 **	0.40 **
FreSHAQ-GR (9-item)		0.93 **

FreSHAQ: Fremantle Shoulder Awareness Questionnaire, SPADI: Shoulder Pain and Disability Index, QuickDASH: Quick Disabilities of the Arm, Shoulder, and Hand, PCS-GR: Pain Catastrophizing Scale, ** *p* < 0.001 (2-tailed).

**Table 8:** Descriptive statistics and reliability coefficients of the 9-item and 6-item FreSHAQ-GR for the sample used (n = 25) for test–retest intra-rater reliability.

***9-Item***	**Day 1**	**Day 2**	**ICC_2,1_ (95% CI)**	**SEM**	**MDC_95%_**
Mean (SD)	11.92 (8.64)	10.96 (7.71)	0.97 (0.91–0.99)	1.31	3.63
Median (IQR)	12.00 (17.00)	12.00 (14.00)			
Grand mean = 11.44.					
***6-Item***	**Day 1**	**Day 2**	**ICC_2,1_ (95% CI)**	**SEM**	**MDC_95%_**
Mean (SD)	9.72 (7.54)	8.96 (7.11)	0.98 (0.94–0.99)	0.94	2.61
Median (IQR)	11.0 (13.0)	10.0 (13.0)			
Grand mean = 9.24.					

## Data Availability

Data are available upon reasonable request.

## Supplementary Materials

The following supporting information can be downloaded at: https://www.mdpi.com/article/10.3390/healthcare11182512/s1, The Fremantle Shoulder Awareness Questionnaire (FreSHAQ).
