# Supplementary material for: Cross-Cultural Adaptation, Reliability, and Validity of the Greek Version of the Fremantle Shoulder Awareness Questionnaire (FreSHAQ-GR) in Patients with Chronic Shoulder Pain
Source: Healthcare (Basel). 2023 Sep 11;11(18):2512. doi: 10.3390/healthcare11182512 (PMC10531340; doi:10.3390/healthcare11182512)
Supplement: Supplementary file 1 [file healthcare-11-02512-s001.zip › healthcare-2551158-supplementary.pdf]

Ερωτηματολόγιο Σωματικής Αυτο-Αντίληψης του Ώμου
The Greek Fremantle Shoulder Awareness Questionnaire (FreSHAQ-GR)

Τα παρακάτω αποτελούν φράσεις που άλλοι ασθενείς μας ανέφεραν σε σχέση με το πως αισθάνονται τον επώδυνο ώμο τους. Χρησιμοποιώντας την ακόλουθη κλίμακα, παρακαλούμε σημειώστε το βαθμό στον οποίο νιώθετε τον ώμο σας κατά τον τρόπο που περιγράφεται παρακάτω **όταν είναι επώδυνος**.

0 = Ποτέ δεν τον αισθάνομαι έτσι
1 = Σπάνια τον αισθάνομαι έτσι
2 = Περιστασιακά / για κάποιο χρονικό διάστημα τον αισθάνομαι έτσι
3 = Συχνά / αρκετό χρονικό διάστημα τον αισθάνομαι έτσι
4 = Πάντα / το μεγαλύτερο χρονικό διάστημα τον αισθάνομαι έτσι

	**Ποτέ**	**Σπάνια**	**Περιστασιακά**	**Συχνά**	**Πάντα**
**1.** Νιώθω τον ώμο που πονάει σαν να μην είναι μέρος του υπόλοιπου σώματός μου					
**2.** Χρειάζεται να συγκεντρωθώ ολοκληρωτικά στον ώμο που πονάει έτσι ώστε να καταφέρω να τον κινήσω όπως θέλω					
**3.** Νιώθω τον ώμο που πονάει μερικές φορές να κινείται ακούσια, χωρίς τον έλεγχο μου					
**4.** Όταν εκτελώ καθημερινές δραστηριότητες, δεν γνωρίζω ακριβώς πόσο κινείται ο ώμος που πονάει					
**5.** Όταν εκτελώ καθημερινές δραστηριότητες, δεν είμαι σίγουρος σε ποια ακριβώς θέση βρίσκεται ο ώμος που πονάει					
**6.** Δεν μπορώ να αντιληφθώ το ακριβές περίγραμμα του ώμου που πονάει					
**7.** Αισθάνομαι τον ώμο που πονάει μεγαλύτερο από το πραγματικό του μέγεθος					
**8.** Αισθάνομαι τον ώμο που πονάει μικρότερο από το πραγματικό του μέγεθος					
**9.** Αισθάνομαι ότι οι ώμοι μου διαφέρουν μεταξύ τους (ως προς το μέγεθος και το σχήμα)					

The Fremantle Shoulder Awareness Questionnaire (FreSHAQ)

Here are some things which other patients have told us about how their painful shoulder feels to them. Using the following scale, please indicate the degree to which your shoulder feels this way **when you are experiencing shoulder pain**.

0 = Never feels like that

1 = Rarely feels like that

2 = Occasionally, or some of the time feels like that

3 = Often, or a moderate amount of time feels like that

4 = Always, or most of the time feels like that

	**Never**	**Rarely**	**Occasionally**	**Often**	**Always**
**1.** My sore shoulder feels as though it is not part of the rest of my body					
**2.** I need to focus all my attention on my sore shoulder to make it move the way I want it to					
**3.** I feel as if my sore shoulder sometimes moves involuntarily, without my control					
**4.** When performing everyday tasks I don't know how much my sore shoulder is moving					
**5.** When performing everyday tasks I am not sure exactly what position my sore shoulder is in					
**6.** I can't perceive the exact outline of my sore shoulder					
**7.** My sore shoulder feels larger than it appears					
**8.** My sore shoulder feels smaller than it appears					
**9.** My shoulders feel different between left and right (in terms of size and shape)					
